# Ocular Hypertension and Glaucoma in United States Army Personnel: Barriers to Detection and Impact on Operational Readiness

**DOI:** 10.7759/cureus.105620

**Published:** 2026-03-21

**Authors:** Samantha Singh, Rishab Majumder, Subeg Singh, Allen Keshishian

**Affiliations:** 1 College of Biosciences and Health Professions, California Health Sciences University College of Osteopathic Medicine, Clovis, USA

**Keywords:** glaucoma, military medicine, occupational health, ocular hypertension, operational readiness, preventive medicine, trauma exposure, vision screening, visual performance

## Abstract

Glaucoma and ocular hypertension rank high among the etiologies of irreversible visual impairment. Their presentation to active-duty United States (US) Army personnel carries unique implications for unit force readiness. Both peripheral and central vision are utilized by soldiers for operational tasks of marksmanship, navigation, and surveillance. Risk factors, including ocular trauma, blast exposure, and systemic corticosteroid use specific to military services, may result in earlier onset or progression of glaucomatous disease. Despite the necessity for soldier visual acuity, routine screenings for ophthalmic disease, including glaucoma and ocular hypertension, remain inconsistent across Army healthcare settings. Limited access to ophthalmologic care in field environments, logistical constraints, and the asymptomatic nature of early disease pose barriers to timely diagnosis. Such untreated cases may directly put individual health and unit readiness at risk, as well as quality of life due to the possibility of vision degradation over time. This narrative review synthesizes literature from PubMed, Google Scholar, and the Cochrane Library between 1999 and 2025 to examine glaucoma and ocular hypertension epidemiology in the US Army. Relevant Department of Defense (DoD) publications, clinical guidelines, and Military Health System (MHS) reports were included in this search. Risk factors, barriers for effective screening, and the evaluation for ocular dysfunction on soldier operational readiness are presented to strengthen screening measures, enhance continued care, and preserve visual performance across the military life cycle for the future.

## Introduction and background

Glaucoma and ocular hypertension are leading causes of irreversible blindness, affecting an estimated 3.2 million people worldwide [1]. These ophthalmologic conditions are extensively studied in the general population, though their projected rise within the United States (US) Army personnel remains under-characterized. US Army service members are exposed to occupational hazards, including blast trauma, barotraumas, high-altitude exposures, and systemic corticosteroid use, which may accelerate the onset or progression of ophthalmic conditions. Soldiers are consistently required to complete events of marksmanship, land navigation, and surveillance, requiring visual acuity, including optimal peripheral and central vision, making undiagnosed progressive ocular disease a threat to organizational mission success and individual performance [2]. Routine screening for ocular hypertension and glaucoma is not uniformly implemented across Army healthcare systems. The asymptomatic nature of early ocular pressure diseases, limited access to ophthalmologic care, and lack of diagnostic tools create barriers to timely detection. Soldiers who receive late diagnoses may suffer permanent visual field loss, compromising their operational capabilities and post-service quality of life. Ocular hypertension in this context refers to elevated intraocular pressure (IOP) without structural or functional damage, whereas early glaucoma involves optic nerve changes with or without visual field loss. Functional visual impairment refers to measurable deficits in vision that impact task performance, which typically occur in more advanced stages. This narrative review examines the prevalence and risk factors for ocular hypertension and glaucoma in the military, identifying systemic and logistical barriers to detection, and evaluates their impact on service member operational readiness. In exploring occupational exposures, we conclude with targeted recommendations to improve timely ocular screenings, enhance disease management, and maintain visual acuity throughout the service member’s career and beyond.

## Review

Methods 

**Figure 1:**
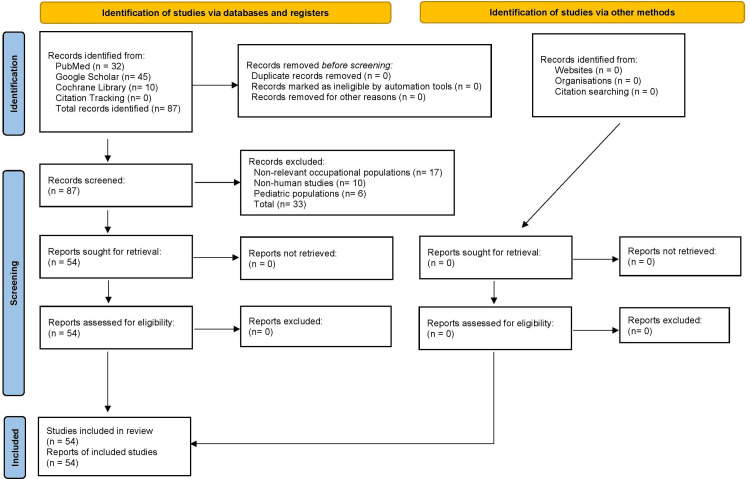
Flow diagram illustrating the literature selection process for the current review.

This narrative review aims to synthesize current literature on the prevalence, risk factors, issues with early detection, and operational impact of ocular hypertension and glaucoma in military personnel. A comprehensive strategy for the usage of relevant studies was employed across major scientific databases, including PubMed, Google Scholar, and the Cochrane Library. Searches include combinations of the following keywords: “glaucoma”, “ocular hypertension”, “vision screening”, “medical readiness”, “intraocular pressure”, “ocular trauma”, “U.S. Army”. Such terminology was selected in order to capture both clinical and occupational dimensions of disease relevant in the military context. Articles published between January 1999 and March 2025 were considered for inclusion. The starting date was chosen to align with the post-Cold War period due to major shifts in US military medical standards and operational environments. Eligible sources within this time frame included Department of Defense (DoD) publications, clinical guidelines, peer-reviewed original research articles, review articles, and military health system (MHS) reports. The consideration of studies with a focus on the military population or that addressed occupational risk factors relevant to service members through blast exposure, barotrauma, or high-G-force environments was prioritized to emphasize operational relevance. Studies were excluded if they focused on non-relevant occupational groups, non-human subjects, or exclusive pediatric populations, as these were considered outside the scope of this review. Citation tracking in the initially selected articles was utilized to gather additional references in addition to hand searches to enhance the comprehensiveness of the search. Given the narrative nature of this review, no meta-analysis or formal quality assessment was performed. Instead, the included literature was thematically analyzed to identify recurring patterns relevant to risk factors, barriers in early detection, and operational implications of glaucoma-related disease within military settings. This approach allowed for a structured synthesis of both clinical evidence and military-specific considerations. A flow diagram is included to illustrate the article selection process (Figure 1); no standardized tools, scales, or scoring systems were used.

Prevalence and military relevance

Ocular hypertension is defined as an increase in IOP above 21 mmHg without structural or functional damage to the optic nerve or visual fields [3]. Glaucoma is an acquired loss of retinal ganglion cells and axons within the optic nerve that results in a progressive loss of vision, with a distinguishing pattern of peripheral vision loss not always associated with elevated IOP [4]. Glaucoma is a leading cause of irreversible blindness worldwide, with an incidence rate of 5.9 cases per 1000 person-years between 2013 and 2017 in the US Army [1]. 

Over the surveillance period, incidence rates of glaucoma diagnoses increased by 75.4% overall, indicating an improved detection among younger service members [1]. Given the asymptomatic nature of glaucoma without explicit testing, the disease is frequently underdiagnosed. With a higher occurrence in younger populations, a 2019 report showed that over 97.6% of these cases were early-stage or borderline, with no reported symptoms [5].

The concern of military screenings excluding IOP or field testing fails to detect vision-compromising conditions until they disqualify members from performing their duties. Vision-critical tasks can include military marches, sustained walking under load. Specifically, the continuous movement without breaks and carrying at least ~10 kg in backpacks has shown effects on IOP [6]. Military marches can include the Valsalva maneuver, an unintentional forceful exhalation exerted during physical strain, which may contribute to transient increases in pressure in the chest and eyes [6]. Other activities such as holding rifles or carrying similar equipment mimic the same effect, which can include transient increases in IOP.

Higher-stakes visual tasks can more prominently impact IOP through their combined effect with physiological stressors. Target recognition, surveillance, and aviation involve complex visual discrimination under pressure, which causes IOP spikes [7]. Aviation, specifically under night conditions, often requires members to wear night-vision goggles (NVG), which can strain the optic nerve more intensely. NVGs present images in monochrome and reduced contrast, which limits depth cues; this requires increased visual adaptation and may contribute to ocular strain [8]. Additional strain may stem from the peripheral compromise, because the physical wearing of NVG limits the range of the head rotation to ~40° vs. ~180° normally, which may further increase visual processing demands [8].

Deployment standards are specific for visual requirements, in that members must not have any glaucoma diagnoses, nor ocular hypertension (eye pressure > 21 mmHg), measured twice on separate days [9]. This automatic disqualification also applies to applicants if they have primary, secondary, or pre-glaucoma, measured by having eye pressure > 21 mmHg, or any damage to the optic nerve/visual field that can suggest early signs of the disease [10].

Risk Factors Specific to Military Service

The pathogenesis of glaucoma and ocular hypertension is influenced by modifiable and nonmodifiable risk factors. Among these risk factors are age, race, family history, and central corneal thickness (CCT) in the general population. Military personnel are subject to additional risks from occupational and environmental exposures [11]. Between training and active combat, military personnel are exposed to a variety of environmental conditions that may play a role in increased risk of ocular hypertension and glaucoma, such as blast waves, high G-forces, high altitudes, ultraviolet radiation, dehydration, and corticosteroid exposure (including systemic, inhaled, and topical forms).

Ocular Trauma and Blast Exposure

Ocular trauma is one of the most prominent vision-threatening injuries that can occur among military personnel. A well-documented cause of glaucoma is often seen through combat, training, accidents, and blunt force impacts. A study of US service members reported 32 ocular injuries per 10,000 personnel per year [12]. Ocular trauma has been observed to be a significant contributor to secondary glaucoma in Army populations [13]. In a retrospective study of US Army hospital discharge records, 77.1 per 100,000 persons were hospitalized for a principal or secondary diagnosis of ocular trauma, many from contusions to the eye from machinery or tools and transport accidents [13].

The exposure to combative blunt force trauma, shrapnel, or accidental injury during training results in exposure to trauma affecting the normal physiology of the eye [14]. Among the unique risk factors in military settings, blast exposures stemming from breaching operations, heavy artillery use, and areas prone to improvised explosive devices (IEDs) are regularly subjected to overpressure waves that can affect multiple organ systems, including the eye [15].

Explosive blasts were reported to be the most common cause of ocular injuries [16]. Exposure to the blasts can result in indirect traumatic optic neuropathy (ITON). The ITONs are associated with significant vision loss through a transient elevation of IOP with retinal ganglion ciliary death and axon degeneration throughout the optic nerve [17]. Results of the blast also induced a 20% IOP increase lasting three days after exposure [17]. These injuries may lead to permanent optic nerve damage and loss of functional vision over time if left untreated.

Different forms of structural damage, including angle recession, hyphema, or trabecular meshwork injury, may lead to chronically elevated intraocular pressure [18]. Angle recession in these situations is defined as the tearing between the muscle layers of the eye's drainage system, usually the iridocorneal angle. Weakening the eye's ability to drain aqueous humor can also cause the mentioned increase in IOP and can further progress to secondary glaucoma if left untreated [19]. Stretching of the internal structures (iridocorneal angle) suddenly and forcefully causes instantaneous and intense pressure fluctuations within the eye. About 7%-9% of these angle recessions develop glaucoma, sometimes long after the initial injury, making screening crucial during this time [20].

Pharmacologic Exposures

Pharmacologic exposures, including the use of topical and systemic corticosteroids, are implicated in pressure elevations [21]. Corticosteroids are routinely prescribed in military care scenarios for conditions including inflammation, dermatologic, respiratory, and musculoskeletal conditions [21]. Administration via systemic, inhaled, or periocular topical corticosteroids may lead to an unnoticed increase in IOP.

Aviation, Diving, and Barotrauma

Aviation and diving roles introduce biomechanical stress factors that can further complicate ocular homeostasis. Pilots experience rapid acceleration and deceleration forces, called high-G exposure, which can result in transient increases in IOP [22]. Exposing pilots to high G-forces (>9 Gz) through aviation demonstrated the CCT to increase by ~10% [22]. This study suggested fluid shifts and/or pressure changes occur in the eye as a direct result of the G-force changes. Similarly, aerospace and gravitational effects through similar forces trigger an almost loss of consciousness (A-LOC) [23]. The A-LOC experience occurs due to the increase in G-force stress, manifesting as acute visual issues. Issues such as tunnel vision or blackouts are due to the reduced blood flow and pressure differential within the eye and blood vessels surrounding it [23]. Divers and airborne soldiers face similar risks associated with barometric pressure changes. Inducing barometric and pressure instability within the eye ultimately alters IOP [24]. Measuring eye pressure in those subjected to increasing altitude up to 2.8 ATA demonstrated a drop in IOP by ~1-1.4 mmHg [24]. Increased ambient pressure lowers IOP, which directly alters fluid dynamics within the eye, critical for long-term IOP stability and its relation to glaucoma risk when exposed to these environments. These combined stressors when experienced chronically can predispose members to long-term IOP dysregulation.

Ultraviolet Radiation and Environmental Extremes

Environmental extremes warrant consideration with prolonged ultraviolet (UV) radiation exposure during outdoor operation settings. These deployment settings can contribute to oxidative stress in ocular tissues, exacerbating optic nerve vulnerability to damage [25]. Through UV exposure, the generation of reactive oxygen species (ROS) also damages the cornea, lens, and retina. The damage contributes to cataracts, glaucoma, and macular degeneration [25].

UV exposure in these working conditions also causes protein oxidation-the buildup of insoluble proteins within the eye. Progressive cataracts and cross-linking in lens fibers in the ocular tissues play a considerable role in the health of the optic nerve and lens [26].

Other field conditions commonly noted are surrounded by high altitudes, exposed to high heat, and ruled by dehydration. Impacting the IOP through changes in plasma osmolality and ocular perfusion can be attributed to dehydration [27]. Plasma osmolality and antidiuretic hormone (ADH) increased while IOP dropped significantly from ~16.7 mmHg to ~13.1 mmHg as a result [27]. In military conditions, the environment is similar with dehydration prominent through extended training exercises, limited water access, and hot working conditions. For service members, such changes may complicate the assessment of IOP, suggesting that hydration be considered during ocular health evaluations.

Demographic and Anatomic Vulnerabilities

Occupational and environmental risks are layered with demographic vulnerabilities, with African American and Hispanic members facing higher baseline risks of glaucoma. African American members are 3-4 times more likely to develop glaucoma than White members; the Hispanic race also shows a similar prevalence (~5%) [28]. With early screening potentially inconsistent already in military groups, African American and Hispanic members at a higher baseline risk may be systematically underdiagnosed for ocular issues.

Individuals with high myopia, thin central cornea, or who have a family history of glaucoma are put at a similar disadvantage. Tracking individuals diagnosed with ocular hypertension and assessing risk factors have shown a greater glaucoma risk in those who demonstrate high myopia (-6D or more) [29]. Thin CCT is a biomarker for the measurement of the true vulnerability of glaucoma progression. Considering ethnicity, African American members average CCT to be 20-30 µm thinner than that of Caucasian individuals [29].

Additionally, given the high rate of myopia in younger service members, it also gives rise to an elevated risk of glaucoma development through underestimation of IOP and increased vulnerability in the condition of the optic nerve. Taking family history into consideration, individuals with a first-degree family member diagnosed with glaucoma had a significantly higher chance of developing glaucoma themselves. This was measured at ~8.5% vs. ~7.3% in those without family history [29]. Early and ongoing ophthalmic surveillance is crucial in these populations, though differences in population characteristics and screening practices may influence applicability to US Army personnel. A consolidated comparison of these vulnerabilities is provided in Table 1.

**Table 1:** Summary of modifiable and nonmodifiable factors contributing to ocular hypertension and glaucoma risk among US military personnel. IOP: intraocular pressure, UV: ultraviolet, CCT: central corneal thickness.

Category	Risk factors	Mechanism/pathophysiology	Operational relevance	Key source
Occupational/environmental	Blast or blunt ocular trauma; barotrauma; high G-forces	Pressure or shockwave injury damaged the trabecular meshwork → reduced aqueous outflow → elevated IOP	Common in breaching, artillery, diving, and aviation operations	Bernardo-Colón et al. (2019) [17]
Pharmacologic	Systemic/topical corticosteroids	Glucocorticoid-induced outflow resistance and trabecular remodeling → increased IOP	Steroids prescribed frequently for respiratory or musculoskeletal issues	Phulke et al. (2017) [21]
Environmental extremes	UV radiation; heat or dehydration; high altitude	Oxidative stress and reduced ocular perfusion → compromise optic nerve function	Frequent during desert or mountain deployments	Ivanov et al. (2018) [25]
Demographic/anatomical	African American or Hispanic ancestry; thin corneal thickness; high myopia; family history	Thin CCT underestimates IOP → optic nerve vulnerability	Targeted for early screening in diverse personnel	Gordon et al. (2002) [29]
Systemic/lifestyle	Metabolic syndrome; hypertension; obesity	Vascular dysregulation and impaired ocular blood flow autoregulation	Prevalent in both active duty and veteran populations	Jiang et al. (2012) [28]

Barriers to detection and diagnosis in Army healthcare

Lack of Routine Screening 

Nowak et al. assessed ~105,000 Polish soldiers during a 12-year period to find that there was no standard practice to screen for IOP nor optic disc assessment during active military service [30]. The screening that was conducted focused almost exclusively on basic visual function and refraction. This could identify refractive errors (hyperopia, myopia, astigmatism) but overlooks early ocular hypertension or glaucomatous changes like optic disc cupping, thinning of the retinal fiber layer, and contrast sensitivity loss [30].

The urgent need for structured screening protocols beyond entry-level exams allows conditions to remain asymptomatic until significant damage occurs. This is seen due to a lack of proper tools. Basic eye exams are conducted using an autorefractor, retinoscopy, or a basic vision chart (Snellen chart). Issues can go unnoticed without proper detection and utilization of more advanced tools: a tonometer for IOP measurement, optic coherence tomography (OCT) for high-resolution imaging of the retinal nerve fiber, or automated visual field testing [31].

The study by Nowak et al. emphasized the lack of essential equipment [30]. With a lack of such tools, there is no proper mechanism in place for early detection that can allow for serious ocular conditions to progress until detected in later, more serious stages.

Limited Accessibility

With operation locations also taking place where there is limited access to ophthalmologists and specialized equipment, a barrier in early detection and timely intervention can be halted [6]. More recently, in 2023, Mazo et al. expanded on this idea by using a hyperbaric chamber to measure IOP changes in military settings [24]. Through their findings, the emphasis on specialized tool usage was deemed successful in detecting and understanding ocular risk. However, the prominence of the understanding that these tools are rare in military settings was also emphasized.

Brigando et al. also reported that accurate diagnoses required repeated IOP measurements and optic nerve head assessment using OCT and visual field testing [32]. With fluctuations in IOP, there can be missed cases or borderline diagnoses, emphasizing the need for multiple tests. Additionally, diurnal variations can further complicate detection, especially in settings without continuous access to tools and the ability to take multiple daily measurements. Such complications are often seen in deployed or underserved military environments [32].

Physiological and Operational Barriers

Effective management of cases also requires long-term follow-ups and patient compliance. Fragmented medical records and poor communication have been noted to potentially enhance treatment disadvantages. Further studies have focused on the challenge in deployed environments to track and maintain ocular hypertension management.

With soldiers solely marching, IOP fluctuates within 30 minutes, showing a rebound effect after exertion (marching) has taken place [6]. Sporadic measurements immediately following activities like this can be inaccurate in describing baseline ocular pressure, which further complicates screening and diagnosis. These findings also showcased the urgent need for portable, rugged diagnostic tools and potentially telemedicine solutions tailored to military deployment in order to overcome accessibility barriers to improve health outcomes in service members [6].

Disease Progression and Missed Diagnoses

Gordon et al. highlighted 9.5% of untreated adults with ocular hypertension outright developed open-angle glaucoma within five years, even though there were no initial symptoms or nerve damage [29]. They also demonstrated that optic disc changes were often detected before visual field defects, confirming that structural damage can occur silently.

Furthermore, the Los Angeles Latino Eye Study found that 75% of individuals with ocular hypertension or open-angle glaucoma previously had been undiagnosed despite measurable IOP or optic nerve changes [33]. Taking these studies into consideration, the silent nature of these diseases already leads to high rates of undiagnosed disease without the implementation of proactive screening in place (IOP checking, optic disc imaging, visual field testing).

The Beijing Eye Study reported ~1.3% of adults to have ocular hypertension with elevated IOP without detectable glaucomatous optic nerve damage or visual field loss [34]. With these findings, the focus on groups including those with metabolic disorders emphasizes the silent progression of issues like glaucoma to be dominant in vulnerable populations like service members. Janak et al. elaborated on this idea through the categorization of military members as vulnerable to metabolic disorders [35]. With a higher prevalence of metabolic syndromes related to vascular issues, ocular hypertension screening becomes even more critical in preventative measures. With lifestyle and occupational factors unique to military service members, the exacerbation of such risks and challenges of a timely diagnosis and management becomes factored in. Through presentation of ocular hypertension without symptoms until after damage, combined with the systemic risk of the asymptomatic nature, detrimental impacts dominate missed cases.

Career Barriers

Career and vision-impacting consequences can occur as a result. With that into consideration, military members may become hesitant to share issues they may be having and hesitate to seek proper care for eye- and health-related issues. Hoge et al. studied this, particularly noting that ~38% of members cited concerns that seeking treatment for their health would harm their career status [36]. One in three members avoided seeking help for mental- or health-related issues solely due to fear of medical separation in the military [36].

The stigma in the study conducted showed a high correlation to the fear of being seen as weak and worries about being non-deployable. Related to ocular hypertension is the concern of losing medical clearance, deployment status, or career advancement should a member be diagnosed. McGuffin et al. further addressed this through the role of stigma and leadership [37]. Military culture can frame illness and medical conditions as weak, fueling the internalized stigma that can discourage help-seeking behavior. Leaders who normalize and support medical care can dismantle the stigma and also help prevent fragmentation in electronic medical records (EMR) and poor follow-up on referrals where medical treatment is needed.

EMR Fragmentation and Follow-Up Gaps

EMRs are crucial to track patient trends and records; a lack of integration within systems and operational devices hinders the unification of patient history [38]. Sluggish hardware and software performance can be lacking, especially in military settings, which can disrupt EMR alignments.

The Government Accountability Office (GAO) report in 2024 confirmed that some military treatment facilities have not transitioned to electronic EMRs, stating that paper records were still in use [39]. The most relevant military EMR system, MHS Genesis, is still being transitioned across units, implying multiple electronic systems are still being utilized in the meantime. Complex operational and technical challenges can be faced due to network issues attributed to field environments. The lack of consistent EMR access and offline or manual paper recordkeeping can manifest further into becoming a challenge to facilitate proper referrals/follow-ups. 

Impact on operational readiness

The impact on operational readiness in military personnel becomes particularly important in light of visual degradation. While early or borderline glaucoma can present without overt symptoms, subtle functional changes may still affect performance under demanding operational conditions. With more advanced glaucoma, impairments can pose a critical threat as core functional capabilities essential for military performance become compromised.

Marksmanship and Target Engagement

One of the potentially threatened roles is marksmanship in both day and night conditions. The assessment of this concept was tested in 2018 with Apimeteetamrong et al. using M16 rifles [2]. The presence of reduced visual contrast sensitivity directly impacted skillset in the task. In night shooting scores, significant differences occurred (mean 5.30 vs. 6.88 daytime, p < 0.001) for overall accuracy [2].

With reduced contrast sensitivity being highly common in glaucoma occurrences, accurate target engagement under variable lighting becomes undermined, and the risk of mission failure in combat environments reliant on visual acuity becomes insufficient.

Night Vision and Dark Adaptation

Demonstration of the concept that glaucoma significantly reduces visual dark adaptation became further validated in a study from 2019 [40]. Ramsey et al. looked at the delayed ability in glaucoma-positive subjects to respond to threats in low-light conditions [40]. They had participants complete a dark adaptation (DA) survey assessing common experiences with nighttime patrols, room-clearing operations, and blackout missions.

The interest was in the difficulty of noticing obstacles at night and adjusting from bright to dark environments. Factor analysis revealed a correlation using regression models between the DA survey and the severity of binocular visual field loss. The higher the score on the DA survey meant a higher perceived difficulty and was tracked as higher in those with glaucoma. Their models achieved a 96.7% accuracy and 99.2% specificity in identifying glaucoma based on survey results [40].

Integration of the DA survey showed the importance of functional visual loss being detectable before severe field deficits occur. Lighting conditions impact these situations as glaucoma patients significantly adapt worse to lighting transitions (glare, adapting to dark environments), with 82% reporting an issue compared to 32% controls without glaucoma [41].

Further elaborating was a study directly validating that glaucoma patients have slower mobility and increased mental workload, an indication of peripheral challenges in dynamic lighting [42]. Measured glare disability was conducted with halo radius testing to generate a glare score and then given conditions with various brightness and dazzling light sources to stimulate glares. Results showed a ~9.3% longer time to complete the course in front of them due to the nonadaptation to lighting conditions presented [42].

Glaucoma-related glare issues slow mobility and increase navigation errors; this can mock military settings with operations following flash blasts. The implication of greater mental processing happens with a higher effort to maintain orientation. Such cognitive overload can significantly decline reaction time and decision quality in the field.

Peripheral Awareness and Situational Responsiveness

Peripheral awareness was shown as 49% more difficult in glaucoma patients [43]. This can translate to the visual hazards in operational surveillance, especially in unpredictable lighting or movement conditions. The functional impairments reported by those impacted by glaucoma can reduce overall responsiveness to sudden visual cues, translating to something as extreme as missed enemy activity or inaccurate threat assessments. The overall mission quality declines without proper addressment of glaucoma-related visual issues, leading to reduced safety and a decline in combat effectiveness.

The silent threat to safety becomes higher in early disease states that do not exclusively present symptoms but still reduce peripheral vision [44]. Affected service members may unknowingly experience visual performance degradation, which can jeopardize both individual and team survival. A delayed diagnosis further leads to performance-limiting symptoms that may not be experienced until mid-mission, posing an unpredictable threat and challenge that could facilitate premature evacuation or reassignment.

Medical Readiness and Deployment Standards

According to the DoD Instruction 6130.03 for Medical Standards, any history of glaucoma, ocular hypertension, or suspicion of glaucoma is automatically disqualifying for military accession [10]. In active-duty personnel, AR 40-501 mandates a Medical Evaluation Board reference if there is no successive treatment for glaucoma.

Failure to preserve adequate visual fields or control disease progression directly can cause reassignment, duty restrictions, or separation. The emphasis on the necessity of early identification and stringent management of glaucoma, preserving deployment readiness across military occupations, is highly regarded in this sense. Visual acuity beyond assessments can reveal unrecognized functional deficits, requiring medication access, regular monitoring, and place-specific duty limitations [45].

The RAND cost-benefit analysis enforces the idea that early detection with an emphasis on a systems-level approach to vision health is essential to sustaining operational effectiveness for visually demanding roles. Beyond glaucoma, chronic conditions can similarly be compared to the complication of deployment and retention planning. Mills et al. showed the comparability of glaucoma to that of chronic diseases like diabetes and osteoporosis [46]. The significant decline in daily physical functionality with progression of the disease is replicated through glaucoma's persistent burden on service members.

Recommendations for improvement

The proposal of solutions through recommendations for improvement in treatment and screening options for glaucoma is crucial for continued care among service members. The treatment of asymptomatic individuals with elevated IOP significantly slowed visual field deterioration, reporting a need for proactive screening [47]. The emphasis becomes higher in high-functioning personnel whose roles depend on vision. Preventing even subclinical visual defects supports long-term combat readiness and can reduce the risk of future medical disqualifications.

Standardized IOP Screening

The reinforced rationale for intertwining standardized IOP screening into annual Periodic Health Assessments with a target on pre-symptomatic diseased individuals can benefit before it ever affects field performance [47]. RAND also performed a cost-benefit analysis under the National Defense Research Institute (NDRI), sponsored by the Office of the Secretary of Defense [45]. RRA2188-1 becomes a defense policy oriented with strategy, carrying an actionable weight for military readiness decisions.

Their analysis directly supported comprehensive eye exams with the inclusion of glaucoma testing through military eye clinics. This would highlight vision screening gaps if basic acuity were evaluated through the importance of expanding vision screening with glaucoma-related testing options.

Increased Optometry and Ophthalmology Support

The increase in optometry support through military facilities can contribute to a significant treatment resource for members. The use of portable tonometry or tele-ophthalmology for field screening can be accurate, patient-focused, and resource-efficient, according to Jesus et al. in a 2025 systematic review [48]. The reduction of travel and clinical reliance while upholding diagnostic integrity is crucial for accurate representation of glaucoma issues in members of military settings.

Being streamlined to specialists and referrals with high-risk individuals in conditions promises operational readiness. The allowance for initial screening and continuous monitoring to be performed remotely/in field settings can promptly identify patient needs and tailor to a more advanced care option. The reduction in delays for diagnosis and treatment ensures appropriate interventions to avoid compromising duty performance or becoming medically disqualified.

Education of Medical Personnel

Sustaining ocular health and combat effectiveness is a priority, and efficient referral systems enhance the community and continuity of care needed while members transfer between service areas. The US Army recognizes the importance of early detection and awareness of glaucoma to members as they affirm the necessity for regular eye examinations to detect the disease in early stages [49].

Healthcare providers being educated about risks and signs of glaucoma to ensure appropriate management are also emphasized as just as important. It becomes equally crucial for the training of personnel to identify indicators like optic nerve cupping and visual field defects.

Integration and Continuity of Care

The integration of ocular health into medical readiness tracking systems allows for better intervention and preservation of vision for operational capabilities. The establishment of clear referral pathways for individuals, especially those with a history of ocular trauma or high-risk individuals, highlights how essential effective glaucoma management should be. The US Navy’s Ophthalmology waiver guide establishes the necessity of mitigating the risk for significant vision impairment [9].

Ensuring personnel remain fit for duty is a strong priority and is upheld through a successful continuity of care. The proposal of strategies aims to preserve vision during active duty and throughout the service member’s lifetime, sustaining the support of long-term operational readiness. These measures emphasize early detection, consistent monitoring, and timely intervention to reduce the risk of vision-related limitations. Table 2 further expands on these ideas.

**Table 2:** Barriers and recommended strategies for early glaucoma detection and management within US Army healthcare settings IOP: intraocular pressure, MHS: military health system, EMR: electronic medical records.

Barrier type	Description/impact	Recommended strategy/improvement	Key source
Screening infrastructure	Limited access to tonometry/visual-field testing in routine exams → early glaucoma missed → reliance on visual-acuity charts only	Integrate standard IOP screening and optic-nerve evaluation into annual periodic health assessments; expand glaucoma testing within military eye clinics	Vardavas et al. (2024) [45]
Accessibility	Shortage of optometry support in remote/deployed settings → delayed referrals → incomplete follow-ups	Expand tele-ophthalmology/portable-tonometry systems to field units; increase optometry staffing within military treatment facilities	Jesus et al. (2025) [48]
Operational physiology	IOP fluctuations during exertion/amplitude shifts → complicated diagnostic accuracy	Establish protocols for field testing and record IOP variations to avoid misdiagnoses	Makateb and Bayat (2021) [6]
Cultural/career concerns	Fear of disqualification or perceived weakness → discouraged to seek care → stigma persists around medical reporting	Leadership-driven education to normalize medical care; integrate confidentiality in ocular reporting	Hoge et al. (2004) [36]
Data and continuity	Fragmented EMR systems/slow MHS GENESIS → missed follow-ups → incomplete data reporting	Accelerate EMR integration; mandate vision-care data uploads and centralized follow-up tracking	US GAO (2024) [39]
Training and awareness	Limited provider familiarity with glaucoma indicators and referral criteria → delayed diagnosis → inconsistent management/treatment	Expand education for providers on early glaucoma; establish clear referral pathways and readiness tracking metrics	US Navy (2025) [9]

## Conclusions

Glaucoma-related ocular hypertension emphasizes the silent threat to the operational effectiveness of military personnel. The unique risks associated with service members can increase disease onset long before detectable symptoms occur. Current screening practices in many military settings can be insufficient due to their limited accessibility to specialized equipment, scattered medical records, and the stigma around medical reporting. This kind of visual impairment can undermine individual performance and unit readiness, especially in roles reliant on precise visual function like marksmanship, surveillance, and aviation. 

Integration of standardized intraocular pressure assessments into annual health evaluations, expanding optometric resources, and improvement of the continuation of care through electronic health systems can close key gaps in current military vision care. Early detection and consistent disease management are critical in preserving deployment eligibility in addition to sustaining combat readiness. Approaching both the structural and cultural barriers to glaucoma detection can preserve long-term visual health, reduce medical disqualifications, and strengthen overall task readiness in the field.
